# Supplementary figures and images for: Probiotic Supplementation and Human Milk Cytokine Profiles in Japanese Women: A Retrospective Study from an Open-Label Pilot Study
Source: Nutrients. 2021 Jun 30;13(7):2285. doi: 10.3390/nu13072285 (PMC8308220; doi:10.3390/nu13072285)

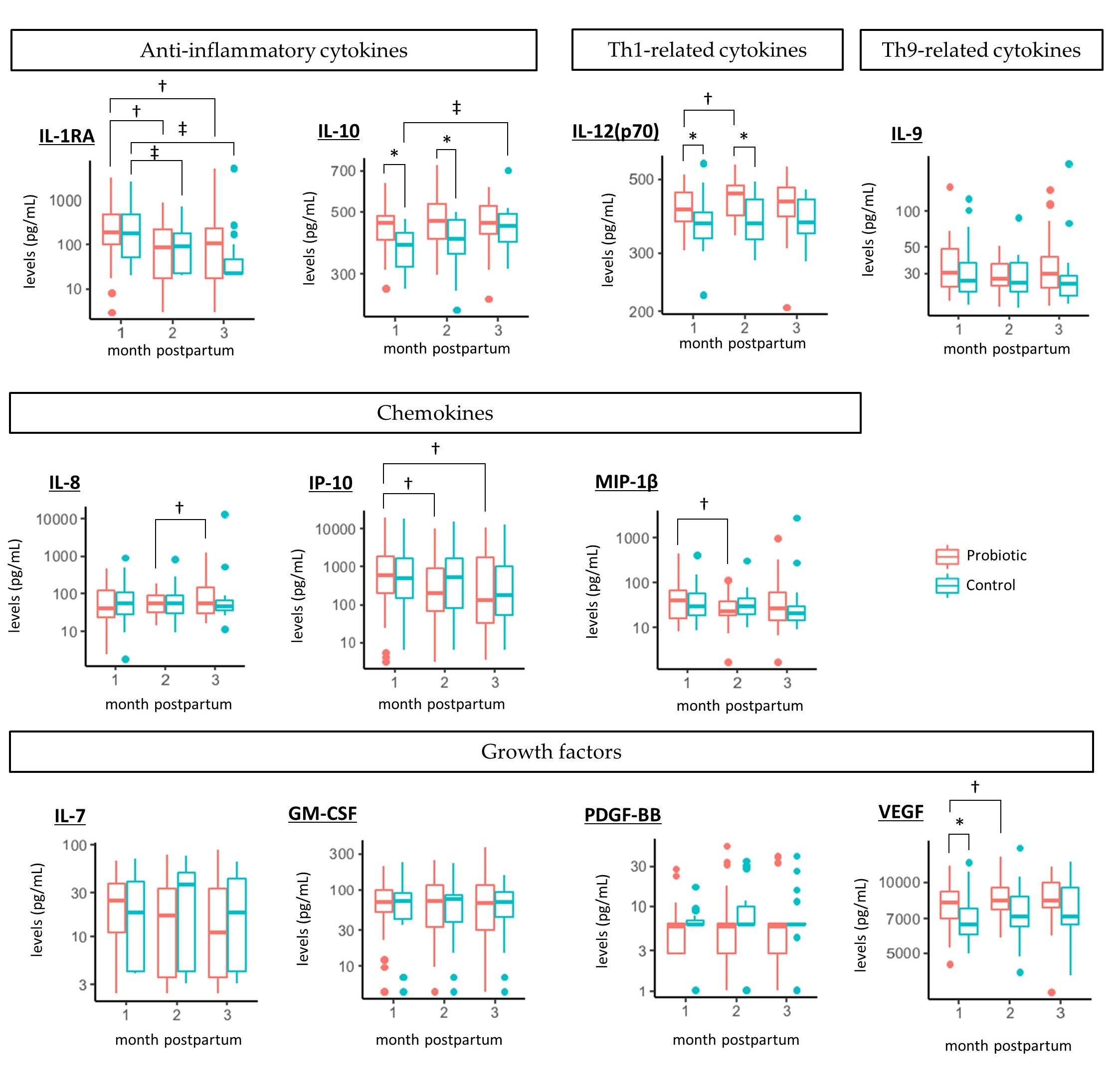

Supplement: Supplementary file 1 [file nutrients-13-02285-s001.zip › Sup.Fig.S1 nutrients-1235253.jpg]

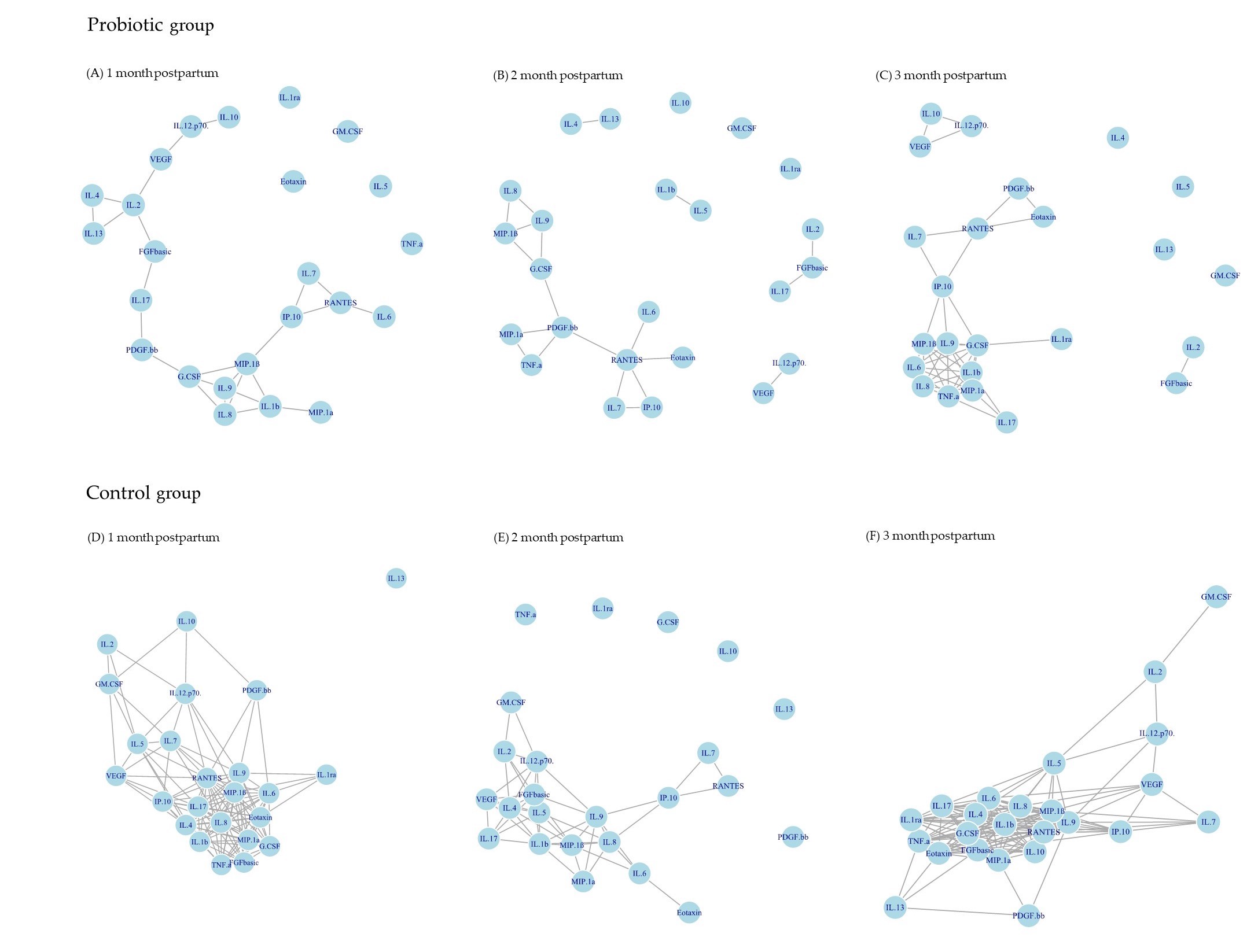

Supplement: Supplementary file 1 [file nutrients-13-02285-s001.zip › Sup.Fig.S2 nutrients-1235253.jpg]

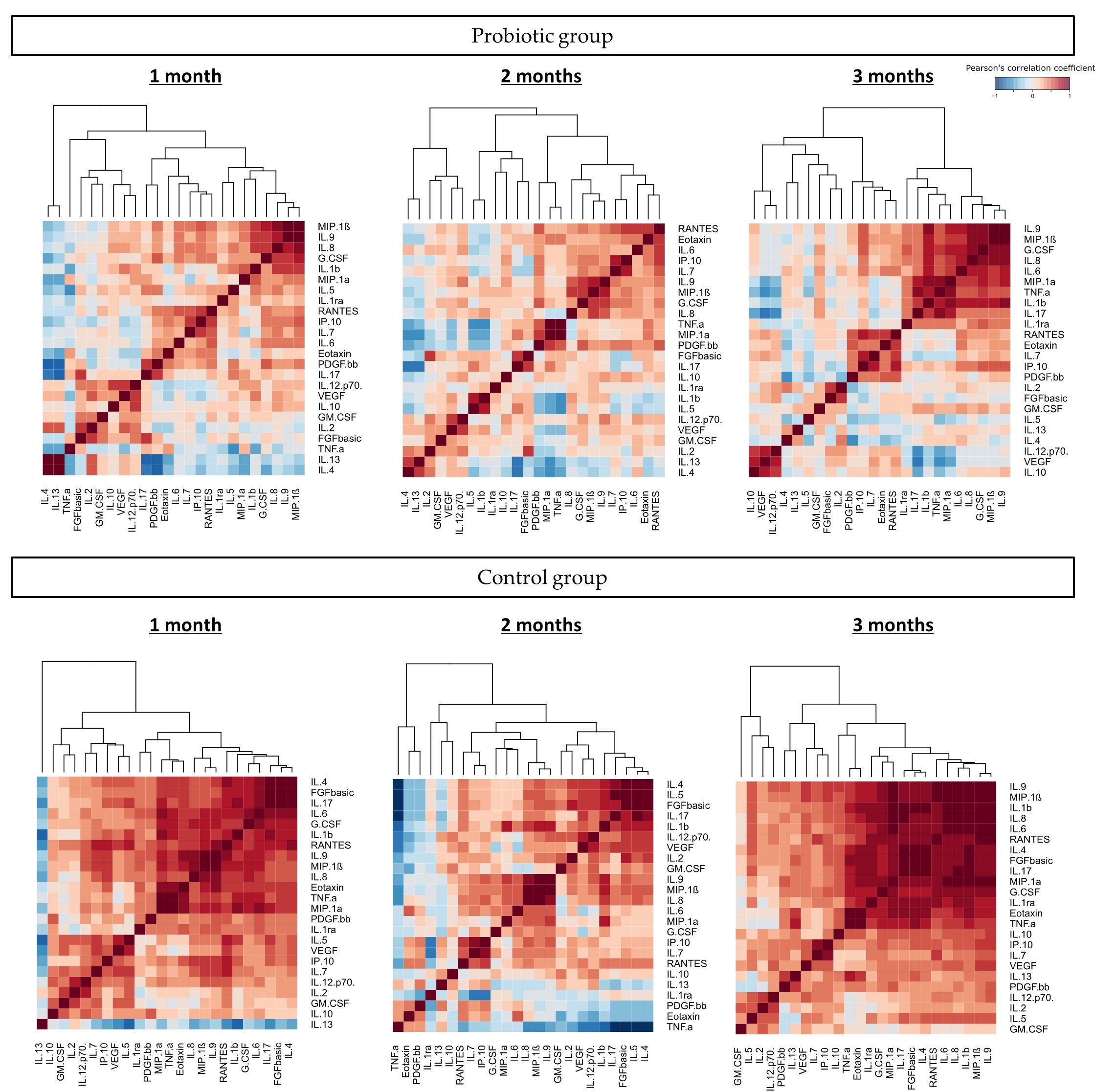

Supplement: Supplementary file 1 [file nutrients-13-02285-s001.zip › Sup.Fig.S3 nutrients-1235253.jpg]
